# A Novel Updated Full-Discretization Method for Prediction of Milling Stability

**DOI:** 10.3390/mi13020160

**Published:** 2022-01-21

**Authors:** Junjin Ma, Yunfei Li, Dinghua Zhang, Bo Zhao, Geng Wang, Xiaoyan Pang

**Affiliations:** 1 School of Mechanical and Power Engineering, Henan Polytechnic University, Jiaozuo 454000, China; iyfhpu369@163.com (Y.L.); zhaob@hpu.edu.cn (B.Z.); wanggeng@hpu.edu.cn (G.W.); 2 Key Laboratory of Contemporary Design and Integrated Manufacturing Technology, Ministry of Education, Northwestern Polytechnical University, Xi’an 710072, China; dhzhang@nwpu.edu.cn; 3 College of Computer Science and Technology, Henan Polytechnic University, Jiaozuo 454000, China; pangxy@hpu.edu.cn

**Keywords:** milling stability, delay-differential equation, computational efficiency, Floquet theory

## Abstract

This paper presents an updated full-discretization method for milling stability prediction based on cubic spline interpolation. First, the mathematical model of the time-delay milling system considering regenerative chatter is represented by a dynamic delay differential equation. Then, in a single tooth passing period, the time is divided into a finite time intervals, the state item and the time-delay item are approximated in each time interval by cubic spline interpolation and third-order Newton interpolation, respectively. Afterward, a transition matrix is constructed to represent the transfer relationship of the teeth in a period. Finally, based on Floquet theory, the milling stability lobes can be obtained. Meanwhile, in order to improve computational efficiency, an optimized method is proposed based on the traditional algorithm and the proposed method has high precision without losing high efficiency. Finally, several milling experiments are conducted to verify the accuracy of the proposed method, and the results show that the predicted results agree well with the experimental results.

## 1. Introduction

Chatter is a serious problem in the milling of a thin-walled workpiece such as aeroengine blades, casings, impellers, blisks etc., which not only reduces the surface quality and production efficiency but also shortens the life of machine spindles and cutters. Therefore, it is necessary to investigate chatter in the milling of thin-walled workpieces for obtaining chatter-free operations. 

Up to now, chatter has been studied by considering the time-varying milling process system by several researchers, including T. Insperger [1,2,3], Chandiramani [4], and Eksioglu [5] et al. From these studies, we found that in the milling process, considerable valuable results on chatter are investigated by a mathematical model of a time-periodic delay differential equation (DDE), and stability lobes diagram can be used to obtain the chatter-free process parameters more accurately.

To investigate machine stability considering regeneration chatter, a considerable number of methods, including analytical methods, numerical methods, and experimental methods, have been proposed to predict the stability lobes diagram (SLD), e.g., the analytical methods [6], the temporal finite element methods [7], the semi-discretization and full-discretization methods [8,9], the time domain numerical simulation methods [10], and the experimental methods [11]. Among the methods mentioned above, the semi-discretization methods and full-discretization methods are widely used due to their efficiency and accuracy.

In recent years, Altintas [12] proposed a zero-order analytical (ZOA) method by the Fourier series average method of dynamic milling force coefficient. Then, Merdol [13] transformed the low radial immersion milling dynamics into an eigenvalue problem by considering the tooth spacing angle and tooth passing frequencies for accurately predicting the stability lobes diagram. The semi-discrete cycloid method based on the nonlinear cutting force milling model was presented by Faassen [14] for investigating the stability of a milling system, which was proved effectively. 

Furthermore, to obtain a high-precision stability lobe diagram, the numerical methods, including the numerical integration method [15,16], Runge–Kutta-based discretization method [17], and precise integration method [18] were developed. Shortly after that, a semi-discrete method (SDM) was proposed by Insperger [19] and then, a full-discretization method (FDM) was presented by Ding et al. [20]. Later on, a second-order FDM method [21], third-order FDM method [22], high-order FDM method [23], Hermite interpolation FDM [24], and the update FDM [25] are successively proposed. It is proved that the accuracy and efficiency of these methods were improved to some extent. However, these extended methods have more complex algorithm structures, which will take more calculation time and obtain the low convergence accuracy to a certain degree. 

Therefore, to obtain a higher convergence rate and computational efficiency, a novel update FDM based on Spline–Newtons interpolation is proposed in this paper. The most important difference of the proposed method compared with the existing methods is that the cubic spline interpolation method was utilized to handle the state item and the third-order Newton interpolation method was used to approximate the time-delay item. The remainder of the paper is organized as follows. In Section 2, a systematic mathematical model and algorithm are described in detail. In Section 3, the rate of convergence estimates of the proposed method is calculated compared with some existing method. In Section 4 and Section 5, two benchmark examples for a one degrees of freedom (DOF) milling model are given to illustrate the accuracy and efficiency of the proposed approach. Some verified experiments are conducted and analyzed in Section 6. Finally, some conclusions are presented.

## 2. Systematic Mathematical Model and Algorithm

**Figure 1:**
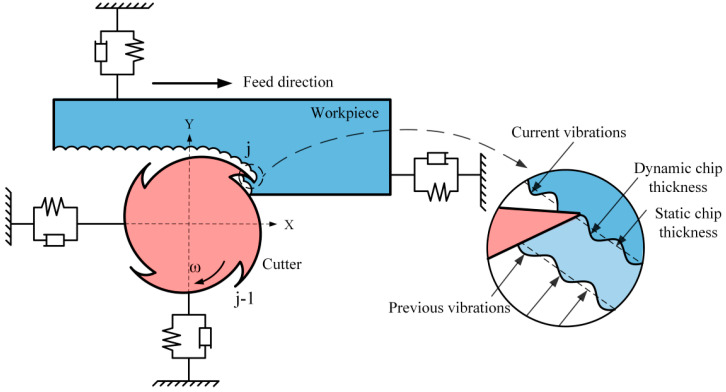
Dynamic model of milling a workpiece.

For a conventional milling process, a schematic diagram of milling a thin-walled section while considering regenerative chatter is given in Figure 1. Without loss of generality, the dynamic model of the milling process system considering the regenerative effect can be expressed by a *n*-dimensional linear time periodic system with a single discrete time delay as follows:

(1) $$\dot{X}(t)=A_{0}X(t)+A(t)[X(t)-X(t-T)]$$

where **A**_0_ is a constant matrix, **A**(*t*) is a time-periodic matrix, and **A**(*t*) = **A**(*t + T*), *T* is the time period which equals to the time delay, and **X**(*t*) is the relative displacement between the cutter and workpiece. In order to solve Equation (1), time period *T* can be equally divided into *m* small-time intervals, and *T* = *mh*, where *m* is an integer and *h* is the range of each interval. Then, the dynamic response of Equation (1) can be obtained by a direct integration method on each time interval *kh* ≤ *t* ≤ (*k* + 1)*h*:

(2) $$X(t)=e^{A_{0}(t-kh)}X(kh)+\int_{kh}^{t}\{e^{A_{0}(t-\varepsilon )}\left[A(\varepsilon )X(\varepsilon )-A(\varepsilon )X(\varepsilon -T)\right]\}d\varepsilon .$$

Let **X**(*kh*) = **X***_k_* with *k* = 0, 1,…, *m*, when *t =* (*k +* 1)*h*, Equation (2) can be equivalently converted to the following form:

(3) $$X_{k+1}=e^{A_{0}h}X_{k}+\int_{0}^{h}e^{A_{0}\varepsilon }\left[A(kh+h-\varepsilon )X(kh+h-\varepsilon )-A(kh+h-\varepsilon )X(kh+h-\varepsilon -T)\right]d\varepsilon$$

Next, the integral term in Equation (3) should be handled. The state item **X**(*kh* + *h −* *ε*) can be approximately represented by cubic spline interpolation using **X***_k+_*_1_, **X***_k_*, **X***_k−_*_1,_ **X***_k−_*_2_. In addition, two other constraints $\dot{X}(kh-2h)$ and $\dot{X}(kh+h)$ are used in cubic spline interpolation. Namely, let **A***_k_* stands for **A**(*kh*):

(4) $$\left\{ \begin{array}{l} \dot{X}(kh-2h+0)=A_{0}X_{k-2}+A_{k-2}(X_{k-2}-X_{k-2-m})\\ \dot{X}(kh+h-0)=A_{0}X_{k+1}+A_{k+1}(X_{k+1}-X_{k+1-m}) \end{array}\right..\;$$

At the time interval [*t_k_*, *t_k_*_+1_], the state item **X**(*kh* + *h −* *ε*) can be approximated by cubic spline interpolation, resulting in:

(5) $$X(kh+h-\varepsilon )=\mu_{1}X_{k+1}^{}+\mu_{2}X_{k}+\mu_{3}X_{k-1}+\mu_{4}X_{k-2}$$

where

(6) $$\mu_{1}=\frac{-11hA_{0}+18I}{15h^{3}}\varepsilon^{3}+\frac{26hA_{0}-33I}{15h^{2}}\varepsilon^{2}-A_{0}\varepsilon +I$$

(7) $$\mu_{2}=\frac{-9I}{5h^{3}}\varepsilon^{3}+\frac{14I}{5h^{2}}\varepsilon^{2}$$

(8) $$\mu_{3}=\frac{4I}{5h^{3}}\varepsilon^{3}-\frac{4I}{5h^{2}}\varepsilon^{2}$$

(9) $$\mu_{4}=\frac{-hA_{0}-3I}{15h^{3}}\varepsilon^{3}+\frac{hA_{0}+3I}{15h^{2}}\varepsilon^{2}.\;$$

The time delay item **X**(*kh* + *h −* *ε* *−* *T*) in Equation (3) can be approximately expressed at four points **X***_k−m+3_*, **X***_k−m+2_*, **X***_k−m_*_+1_, **X***_k_*_−*m*_ by the third-order Newton interpolation method as follows:

(10) $$X(kh+h-\varepsilon -T)=\lambda_{1}X_{k-m}^{}+\lambda_{2}X_{k-m+1}+\lambda_{3}X_{k-m+2}+\lambda_{4}X_{k-m+3}$$

where

(11) $$\lambda_{1}=\frac{\varepsilon^{3}}{6h^{3}}+\frac{\varepsilon^{2}}{2h^{2}}+\frac{\varepsilon }{3h}$$

(12) $$\lambda_{3}=\frac{\varepsilon^{3}}{2h^{3}}+\frac{\varepsilon^{2}}{2h^{2}}-\frac{\varepsilon }{h}$$

(13) $$\lambda_{3}=\frac{\varepsilon^{3}}{2h^{3}}+\frac{\varepsilon^{2}}{2h^{2}}-\frac{\varepsilon }{h}$$

(14) $$\lambda_{4}=\frac{-\varepsilon^{3}}{6h^{3}}+\frac{\varepsilon }{6h}.\;$$

Subsequently, the time-periodic item **A**(*kh* + *h* − *ε*) in Equation (3) can be expressed by linear interpolation which using points **A**(*kh*) and **A**(*kh* + *h*), and:

(15) $$A(kh+h-\varepsilon )=A_{u}+A_{v}\varepsilon$$

where

(16) $$A_{u}=A_{k+1}$$

(17) $$A_{v}=(A_{k}-A_{k+1})/h.\;$$

Then, substituting Equation (5), Equation (10), and Equation (15) into Equation (3) leads to the interpolated item **X**(*kh* + *h *− *ε*), **X**(*kh* + *h* − *ε − T*) and **A**(*kh* + *h *− *ε*) are taken into Equation (3), and the DDE is approximated by an ordinary differential equation (ODE), which can be simplified as follows:

(18) $$\begin{array}{cc} P_{k+1}X_{k+1}= & P_{k}X_{k}+P_{k-1}X_{k-1}+P_{k-2}X_{k-2}+P_{k-m+3}X_{k-m+3}\\ & +P_{k-m+2}X_{k-m+2}+P_{k-m+1}X_{k-m+1}+P_{k-m}X_{k-m} \end{array}$$

where

(19) $$P_{k+1}=I-(a_{1}A_{u}+a_{2}A_{v})$$

(20) $$P_{k}=\Phi_{0}+(b_{1}A_{u}+b_{2}A_{v})$$

(21) $$P_{k-1}=c_{1}A_{u}+c_{2}A_{v}$$

(22) $$P_{k-2}=d_{1}A_{u}+d_{2}A_{v}$$

(23) $$P_{k-m+3}=e_{1}A_{u}+e_{2}A_{v}\;$$

(24) $$P_{k-m+2}=f_{1}A_{u}+f_{2}A_{v}$$

(25) $$P_{k-m+1}=g_{1}A_{u}+g_{2}A_{v}$$

(26) $$P_{k-m}=h_{1}A_{u}+h_{2}A_{v}.\;$$

Define:

(27) $$\Phi_{0}=e^{A_{0}h}$$

(28) $$\Phi_{1}=\int_{0}^{h}e^{A_{0}\varepsilon }d\varepsilon =A_{0}^{-1}(\Phi_{0}-I)$$

(29) $$\Phi_{2}=\int_{0}^{h}\varepsilon e^{A_{0}\varepsilon }d\varepsilon =A_{0}^{-1}(h\Phi_{0}-\Phi_{1})$$

(30) $$\Phi_{3}=\int_{0}^{h}\varepsilon^{2}e^{A_{0}\varepsilon }d\varepsilon =A_{0}^{-1}(h^{2}\Phi_{0}-2\Phi_{2})$$

(31) $$\Phi_{4}=\int_{0}^{h}\varepsilon^{3}e^{A_{0}\varepsilon }d\varepsilon =A_{0}^{-1}(h^{3}\Phi_{0}-3\Phi_{3})$$

(32) $$\Phi_{5}=\int_{0}^{h}\varepsilon^{4}e^{A_{0}\varepsilon }d\varepsilon =A_{0}^{-1}(h^{4}\Phi_{0}-4\Phi_{4}).\;$$

In addition, the coefficients in Equations (19)–(26) can be expressed as:

(33) $$a_{1}=\frac{-11hA_{0}+18I}{15h^{3}}\Phi_{4}+\frac{26hA_{0}-33I}{15h^{2}}\Phi_{3}-A_{0}\Phi_{2}+\Phi_{1}$$

(34) $$a_{2}=\frac{-11hA_{0}+18I}{15h^{3}}\Phi_{5}+\frac{26hA_{0}-33I}{15h^{2}}\Phi_{4}-A_{0}\Phi_{3}+\Phi_{2}$$

(35) $$b_{1}=\frac{-9}{5h^{3}}\Phi_{4}+\frac{14}{5h^{2}}\Phi_{3}$$

(36) $$b_{2}=\frac{-9}{5h^{3}}\Phi_{5}+\frac{14}{5h^{2}}\Phi_{4}$$

(37) $$c_{1}=\frac{4}{5h^{3}}\Phi_{4}-\frac{4}{5h^{2}}\Phi_{3}$$

(38) $$c_{2}=\frac{4}{5h^{3}}\Phi_{5}-\frac{4}{5h^{2}}\Phi_{4}$$

(39) $$d_{1}=\frac{-hA_{0}-3I}{15h^{3}}\Phi_{4}+\frac{hA_{0}+3I}{15h^{2}}\Phi_{3}$$

(40) $$d_{2}=\frac{-hA_{0}-3I}{15h^{3}}\Phi_{5}+\frac{hA_{0}+3I}{15h^{2}}\Phi_{4}$$

(41) $$e_{1}=\frac{1}{6h^{3}}\Phi_{4}-\frac{1}{6h}\Phi_{2}$$

(42) $$e_{2}=\frac{1}{6h^{3}}\Phi_{5}-\frac{1}{6h}\Phi_{3}$$

(43) $$f_{1}=\frac{-1}{2h^{3}}\Phi_{4}-\frac{1}{2h^{2}}\Phi_{3}+\frac{1}{h}\Phi_{2}$$

(44) $$f_{2}=\frac{-1}{2h^{3}}\Phi_{5}-\frac{1}{2h^{2}}\Phi_{4}+\frac{1}{h}\Phi_{3}$$

(45) $$g_{1}=\frac{1}{2h^{3}}\Phi_{4}+\frac{1}{h^{2}}\Phi_{3}-\frac{1}{2h}\Phi_{2}-\Phi_{1}$$

(46) $$g_{2}=\frac{1}{2h^{3}}\Phi_{5}+\frac{1}{h^{2}}\Phi_{4}-\frac{1}{2h}\Phi_{3}-\Phi_{2}$$

(47) $$h_{1}=\frac{-1}{6h^{3}}\Phi_{4}-\frac{1}{2h^{2}}\Phi_{3}-\frac{1}{3h}\Phi_{2}$$

(48) $$h_{2}=\frac{-1}{6h^{3}}\Phi_{5}-\frac{1}{2h^{2}}\Phi_{4}-\frac{1}{3h}\Phi_{3}.\;$$

Then, if the matrix **P***_k_*_+1_ in Equation (18) is nonsingular, Equation (18) can be given by:

(49) $$\begin{array}{ll} X_{k+1}= & P_{k+1}^{-1}P_{k}X_{k}+P_{k+1}^{-1}P_{k-1}X_{k-1}+P_{k+1}^{-1}P_{k-2}X_{k-2}+P_{k+1}^{-1}P_{k-m+3}X_{k-m+3}\\ & +P_{k+1}^{-1}P_{k-m+2}X_{k-m+2}+P_{k+1}^{-1}P_{k-m+1}X_{k-m+1}+P_{k+1}^{-1}P_{k-m}X_{k-m} \end{array}$$

According to Equation (49), a discrete map could be defined as:

(50) $$QN_{k+m}=RN_{k}$$

where

(51) $$N_{k}={\left[X_{k-m},X_{k-m+1},\cdots ,X_{k-1},X_{k}\right]}^{T}.\;$$

In addition, **Q** and **R** can be expressed as:

(52) $$R=\left[\begin{array}{ccccccccccc} 0 & 0 & 0 & 0 & 0 & 0 & \cdots & 0 & 0 & 0 & I\\ P_{k,m} & P_{k,m-1} & P_{k,m-2} & P_{k,m-3} & 0 & 0 & \cdots & 0 & P_{k,-2} & P_{k,-1} & 0\\ 0 & P_{k+1,m} & P_{k+1,m-1} & P_{k+1,m-2} & P_{k+1,m-3} & 0 & \cdots & 0 & 0 & P_{k+1,-2} & 0\\ 0 & 0 & P_{k+2,m}\; & P_{k+2,m-1} & P_{k+2,m-2} & P_{k+2,m-3} & \cdots & 0 & 0 & 0 & 0\\ \vdots & \vdots & \vdots & \vdots & \vdots & \vdots & \ddots & \vdots & \vdots & \vdots & \vdots \\ 0 & 0 & 0 & 0 & 0 & 0 & \cdots & P_{k+m-3,-2} & P_{k+m-3,-1} & P_{k+m-3,0} & P_{k+m-3,1}\\ 0 & 0 & 0 & 0 & 0 & 0 & \cdots & 0 & P_{k+m-2,-2} & P_{k+m-2,-1} & P_{k+m-2,0}\\ 0 & 0 & 0 & 0 & 0 & 0 & \cdots & 0 & 0 & P_{k+m-1,-2} & P_{k+m-1,-1} \end{array}\right]$$

(53) $$Q=\left[\begin{array}{ccccccccccc} I & 0 & 0 & 0 & \cdots & 0 & 0 & 0 & 0 & 0 & 0\\ P_{k,0} & P_{k,1} & 0 & 0 & \cdots & 0 & 0 & 0 & 0 & 0 & 0\\ P_{k+1,-1} & P_{k+1,0} & P_{k+1,1} & 0 & \cdots & 0 & 0 & 0 & 0 & 0 & 0\\ P_{k+2,-2} & P_{k+2,-1} & P_{k+2,0} & P_{k+2,1} & \cdots & 0 & 0 & 0 & 0 & 0 & 0\\ \vdots & \vdots & \vdots & \vdots & \ddots & \vdots & \vdots & \vdots & \vdots & \vdots & \vdots \\ 0 & 0 & 0 & 0 & \cdots & P_{k+m-3,-2} & P_{k+m-3,-1} & P_{k+m-3,0} & P_{k+m-3,1} & 0 & 0\\ 0 & 0 & 0 & 0 & \cdots & 0 & P_{k+m-2,-2} & P_{k+m-2,-1} & P_{k+m-2,0} & P_{k+m-2,1} & 0\\ 0 & -P_{k+m-1,m-2} & -P_{k+m-1,m-3} & 0 & \cdots & 0 & 0 & P_{k+m-1,-2} & P_{k+m-1,-1} & P_{k+m-1,0} & P_{k+m-1,1} \end{array}\right]$$

It is clear that **Q** and **R** are both a (2*m* + 2) × (2*m* + 2) dimensional matrix. Therefore, the transition matrix **V** in a single tooth passing period can be defined as:

(54) $$V=Q^{-1}R.\;$$

Now, according to Floquet theory [26], the stability of the system can be determined by judging whether the modulus of the eigenvalues of the transition matrixes are less than 1 or not. If not, the system is unstable, otherwise, it will be stable.

Remark: If **P***_k_*_+1_ is singular, the processing method in Ref. [20] can be utilized. From Equations (52) and (53), it can be seen that 8*m* variables need to be calculated complicatedly compared with the first-order and second-order FDM, which leads to the increase of calculating time. To enhance the calculation efficiency, the traditional algorithms compressed the 2*m* + 2 dimensional matrix into a *m* + 2 dimensional matrix, and calculated the eigenvalues of the transition matrix in one period in the whole region, then, the stability boundary is drawn. However, a novel algorithm is proposed to obviously improve computational efficiency. It is well known that the machining process is stable below the boundary of the SLD and is unstable on the upper boundary of the SLD, while on the stable boundary the eigenvalue of the transition matrix is 1, represented by the spindle speed and depth of cut. According to the constraints of the spindle speed and depth of cut, the modulus of the transition matrix eigenvalues are calculated and judged with 1. If the value is more than 1, the algorithm stops, otherwise it continues, which is only the modulus of transition matrix eigenvalues that are less than 1 and calculated for the stability boundary, which can greatly improve the computational efficiency by reducing the calculation of the eigenvalues in the unstable region.

## 3. Rate of Convergence Estimates

To verify the fast convergence rate of the proposed method, a one DOF dynamic milling system is selected, and the proposed method is compared to the 0th SDM, 1st FDM, 2nd FDM, 3rd FDM, and Hermite FDM.

The rate of convergence can be clearly determined by the local discretization error (LDE). As stated in the literature [19], for the 0th SDM, the LDE is ***O***(*h*^2^). The LDE of FDM with the 1st, 2nd, 3rd, and Hermite are ***O***(*h*^2^), ***O***(*h*^3^), ***O***(*h*^4^), and ***O***(*h*^3^) [24,27], respectively. For the proposed method, the LDE is ***O***(*h*^4^).

All operations are from the same computer environment: Matlab 2018b, Inter(R) Core(TM) i5-4210H CPU @ 2.90 GHz 2.90 GHz. The milling system parameters are derived from the Ding [20]: The damping ratio *ζ*, model mass *m_t_*, and natural frequency *w_n_* are 0.011, 0.03993 kg, and 1844 Hz, respectively. The cutter has two flutes. The cutting force coefficients are *K_tc_* = 6 × 10^8^ and *K_rc_* = 2 × 10^8^.

The spindle speed *n* is selected as 5000 rpm, and the axial depths of cut *a_p_* is selected as 0.1 mm, 0.2 mm, 0.5 mm, and 0.80 mm, respectively. The exact eigenvalues $\left|\mu_{0}\right|$ corresponding to different axial depths of cut are 0.7368, 0.8192, 1.0726, and 1.2880, respectively. The LDE can be known as the absolute value of difference between the current eigenvalue |μ| and exact eigenvalue $\left|\mu_{0}\right|$.

**Figure 2:**
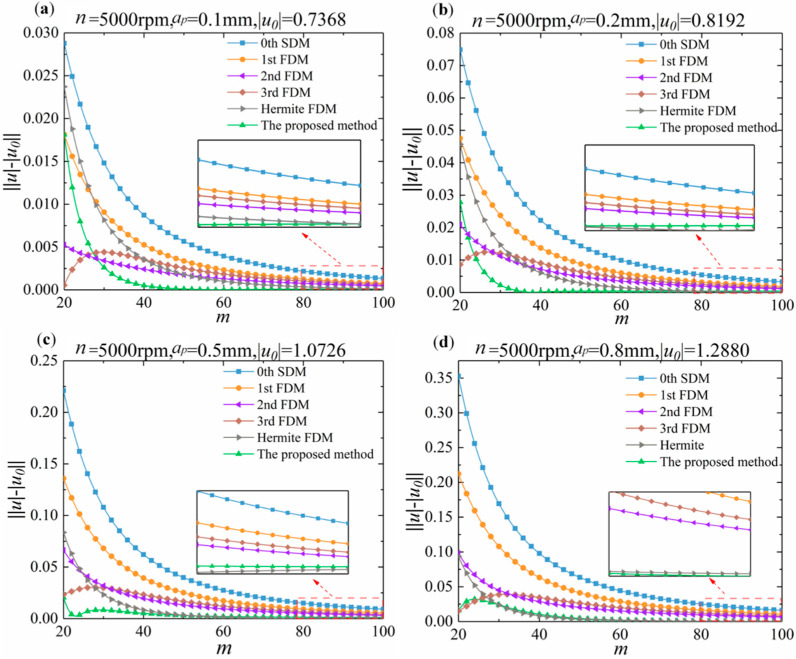
The convergence rate of eigenvalues for different discrete numbers *m* for the 0th SDM, 1st FDM, 2nd FDM, 3rd FDM, Hermite FDM, and the proposed method. (**a**) Axial depth of cut *a_p_* is 0.1 mm, exact eigenvalue |**μ**_0_| is 0.7368. (**b**) Axial depth of cut *a_p_* is 0.2 mm, exact eigenvalue |**μ**_0_| is 0.8192. (**c**) Axial depth of cut *a_p_* is 0.5 mm, exact eigenvalue |**μ**_0_| is 1.0726. (**d**) Axial depth of cut *a_p_* is 0.8 mm, exact eigenvalue |**μ**_0_| is 1.2880.

As shown in Figure 2, the LDE of the 0th SDM, 1st FDM, 2nd FDM, 3rd FDM, Hermite FDM, and the proposed method are analyzed. From Figure 2, it can be clearly seen that the proposed method has a faster convergence rate. It should be mentioned that the proposed method is able to converge to a sufficient accuracy when the discrete number *m* is small.

## 4. Computational Accuracy Analysis

Under low and high immersion ratio conditions, the effectiveness of the proposed method is verified in terms of both computational efficiency and accuracy of milling stability prediction by comparing with other methods. The modal parameter selection is consistent with Section 3 of this paper. Equation (1) is the dynamic state-space model of the milling system, where constant matrix **A**_0_ and time-periodic matrix **A**(*t*) can be express as:

(55) $$A_{0}=\left[\begin{array}{ll} -\zeta w_{n} & \;1/m_{t}\\ m_{t}{\left(\zeta w_{n}^{}\right)}^{2}-m_{t}w_{n}^{}{}^{2} & -\zeta w_{n}^{} \end{array}\right]$$

(56) $$A(t)=\left[\begin{array}{ll} 0 & 0\\ -a_{p}h(t) & 0 \end{array}\right]$$

(57) $$h(t)=\sum_{j=1}^{N}g(\varphi_{j}(t))\sin(\varphi_{j}(t))(K_{tc}\cos(\varphi_{j}(t))+K_{rc}\sin(\varphi_{j}(t)))$$

(58) $$\varphi_{j}(t)=\frac{2\pi n}{60}t+(j-1)\frac{2\pi }{N}$$

(59) $$g(\varphi_{j}(t))=\left\{ \begin{array}{ll} 1 & \varphi_{st}<\varphi_{j}(t)<\varphi_{ex}\\ 0 & otherwise \end{array}\right.$$

where *φ_j_*(*t*) is the angular position of the *j*-th cutter tooth, and *φ_st_* and *φ_ex_* are the starting and exiting edge positions of the tool in contact with the workpiece, respectively. For down-milling, *φ_st_* = arccos(2*a/D* − 1) and *φ_ex_* = π; for up-milling, *φ_st_* = 0 and *φ_ex_* = arccos(1 − 2*a/D*), where *a/D* is the radial immersion ratio.

**Figure 3:**
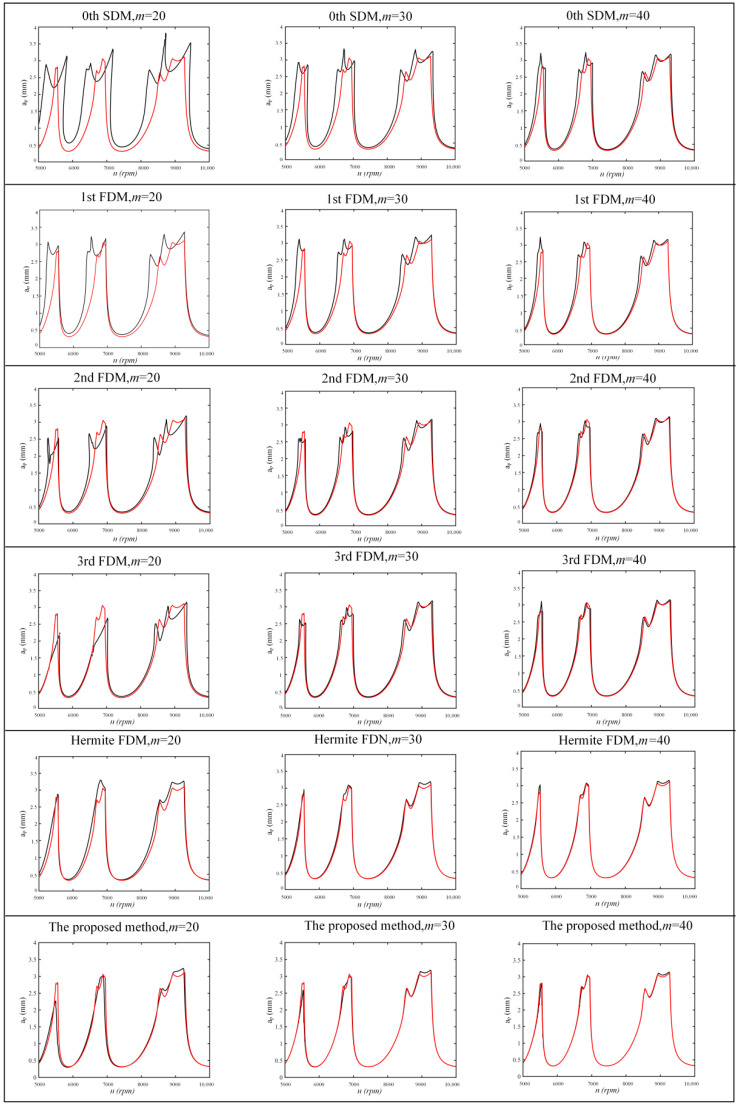
A comparison of computational accuracy in *a/D* = 1.

**Figure 4:**
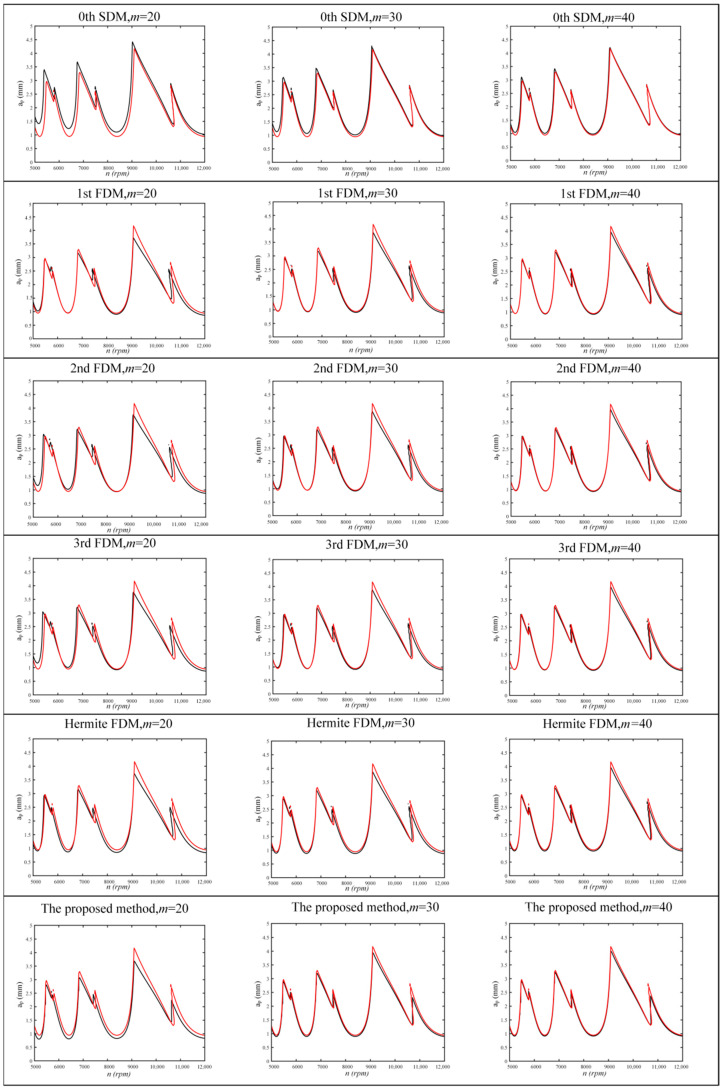
A comparison of computational accuracy in *a/D* = 0.1.

For *a/D* = 1, all methods are calculated over a 200 × 100-sized grid of parameters under the condition of *n* = 5000–10,000 rpm and *a_p_* = 0–4 mm. For *a/D* = 0.1, all methods are calculated over a 200 × 100-sized grid of parameters under the condition of *n* = 5000–12,000 rpm and *a_p_* = 0–5 mm. The SLDs for *a/D* = 1 and *a/D* = 0.1 are shown in Figure 3 and Figure 4. The red curves in Figure 3 and Figure 4 are the reference curves, calculated by the Hermite FDM at the discrete number *m* = 100. It can be seen that the proposed method has a high accuracy both at a low and high radial immersion ratio. In particular, the 0th SDM, 1st FDM, 2nd FDM, 3rd FDM, and Hermite FDM all have large errors with the reference curve when *m* = 20 at the *a/D* = 1. However, the proposed method almost coincides with the reference curve. For *a/D* = 0.1, the 1st FDM agrees best with the reference curve when *m* = 20, and the proposed method agrees with the reference curve immediately as *m* increases.

## 5. Computational Efficiency Analysis

**Figure 5:**
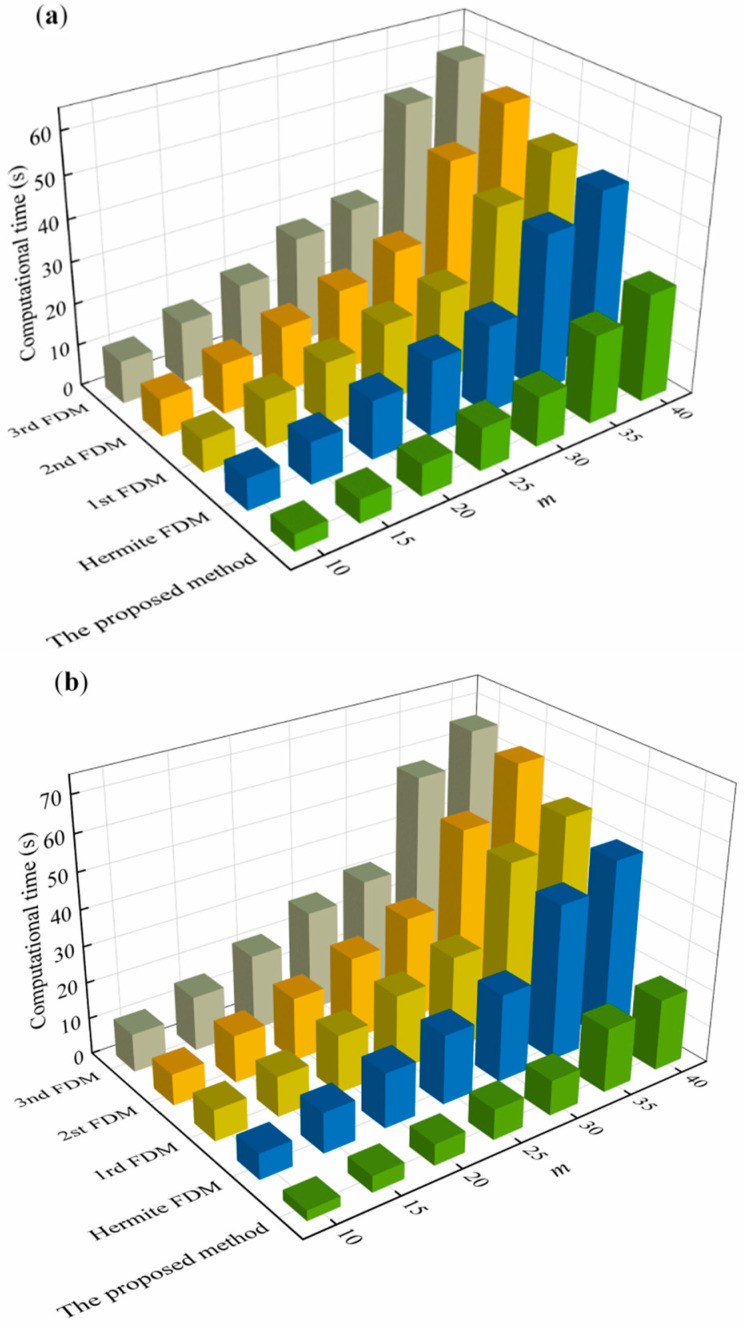
Comparison of calculated time among the 0th SDM, 1st FDM, 2nd FDM, 3rd FDM, Hermite FDM, and the proposed method with different radial immersion ratios. (**a**) Calculated time at *a/D* = 0.1 and (**b**) Calculated time at *a/D* = 1.

To verify the computational efficiency of the proposed method, the time required for the computation of the FDM in Section 4 for different discrete numbers *m* is discussed. The time required for the calculation is shown in Figure 5. From Figure 5, it can be seen that the proposed method has a faster computational efficiency compared to other methods. When *a/D* = 1, the proposed method saves an average of 69.2%, 73.3%,75.4%, and 66.7% of time compared to the 1st FDM, 2nd FDM, 3rd FDM, and Hermite FDM, respectively. When *a/D* = 0.1, the proposed method saves an average of 53.3%, 58.8%, 63.8%, and 47.5% of time compared to the 1st FDM, 2nd FDM, 3rd FDM, and Hermite FDM, respectively. It can be seen that the proposed method has a higher computational linear efficiency when *a/D* = 1. The main reason is that when *a/D* = 1, the contact time between the cutter and workpiece is at a maximum, while the transfer matrix Equation (54) needs to be calculated multiple times, and the proposed method saves the calculation time required for the stability region.

## 6. Verification

**Figure 6:**
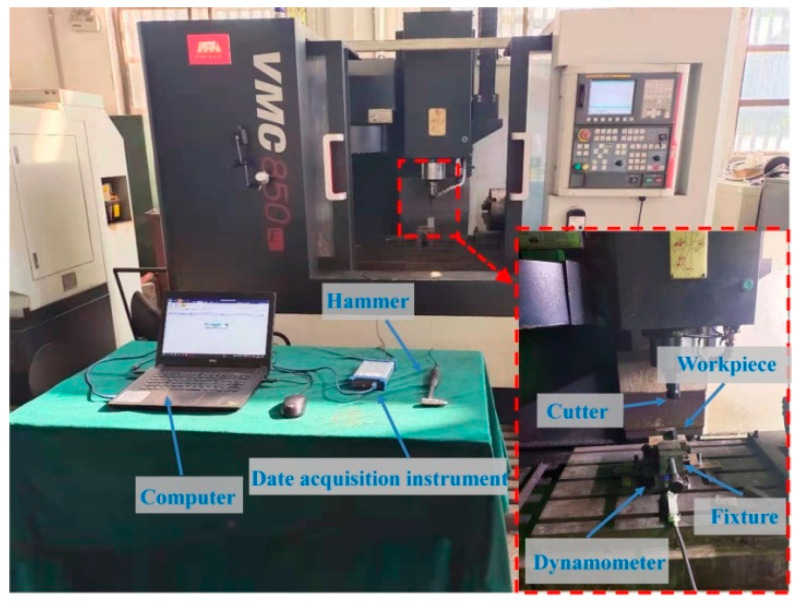
Configuration of the experiments.

To verify the effectiveness of the proposed method in the milling of the thin-walled plate, some experiments are conducted in this section. The dimension of the plate used in modal test and machining experiments is 80 × 40 × 3 mm, and all experiments were carried out on the three-axis milling center (VMC-850E), which is shown in Figure 6. The material properties of the workpiece and the cutter parameters are given in Table 1.

### 6.1. Cutting Force Coefficients Calibration

For cutting force coefficients calibrated, as is known to all, when full-immersion milling (slot milling) is used, the average milling forces are expressed as:

(60) $$\left\{ \begin{array}{l} {\bar{F}}_{x}=-\frac{Na}{4}K_{rc}f-\frac{Na}{\pi }K_{re}\\ {\bar{F}}_{y}=\frac{Na}{4}K_{tc}f+\frac{Na}{\pi }K_{te} \end{array}\right..$$

Then, for slot milling, five groups of full-immersion milling experiments were carried out. The machining parameters are the spindle speed 1000 rpm, axial depth of cut at 0.5 mm, and feed rate at 40 mm/min, 80 mm/min, 120 mm/min, 160 mm/min, and 200 mm/min. 

Therefore, the average milling forces at each feed rate are measured by Kistler9257B, and the cutting-edge components are estimated by a linear regression of the accumulated data. Next, based on the literature [28], the cutting force coefficients are evaluated as *K_tc_* = 1120.8 N/mm^2^, *K_rc_* = 2285.6 N/mm^2^, *K_te_* = 9.16 N/mm, and *K_re_* = 13.21 N/mm.

### 6.2. Modal Parameters Identification

An impact experiment is conducted for obtaining the modal parameters of the thin-walled workpiece. The modal parameters of the milling system are obtained by an acquisition instrument DH5981, acceleration sensors (Ref. sensitivity 10.25 mV/g), and modal hammer (500 N).

**Figure 7:**
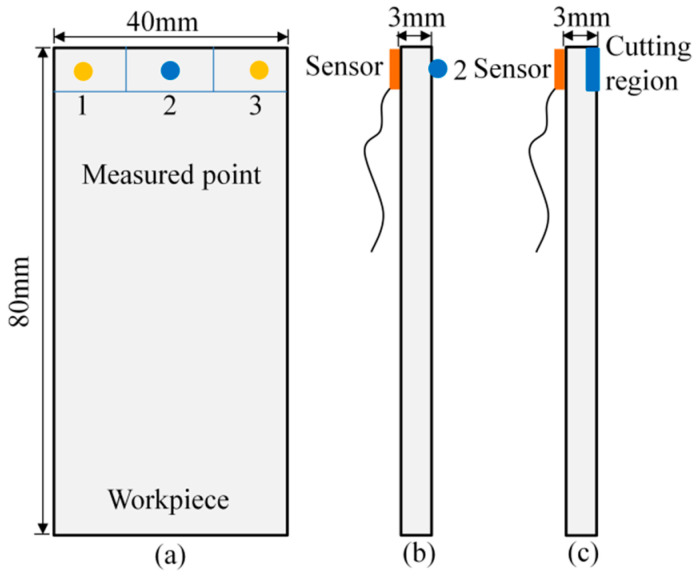
(**a**) Distributed points 1, 2, and 3 on the thin-walled plate for impact tests. (**b**) Position of the sensor and point 2 for measuring responses. (**c**) Position of the sensor and cutter region for machining.

In tests, for a different measured position on the workpiece, the dynamic response is different. Therefore, considering the clamping constraints, the impact measured points 1, 2, and 3 distributed on the thin-walled plate are shown in Figure 7a. Point 1 and point 3 are symmetric with respect to point 2, and point 2 locates the middle of the thin-walled plate edge. Next, all the vibration responses on the different measured points are obtained, and according to the experimental results, we found that the vibration response at point 1 is the same as that at point 3. In addition, considering the unstable state in the cut-in and cut-out region, the representative point 2 are chosen for measuring responses, as shown in Figure 7b,c, and the experimental setup is shown in Figure 6. Therefore, the modal parameters in point 2 are identified and listed in Table 2.

### 6.3. Machining Tests

**Figure 8:**
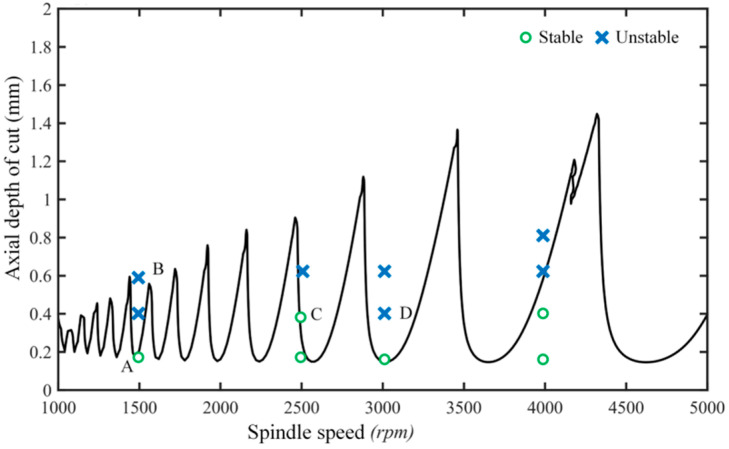
Stability lobe diagrams at the measured point 2.

**Figure 9:**
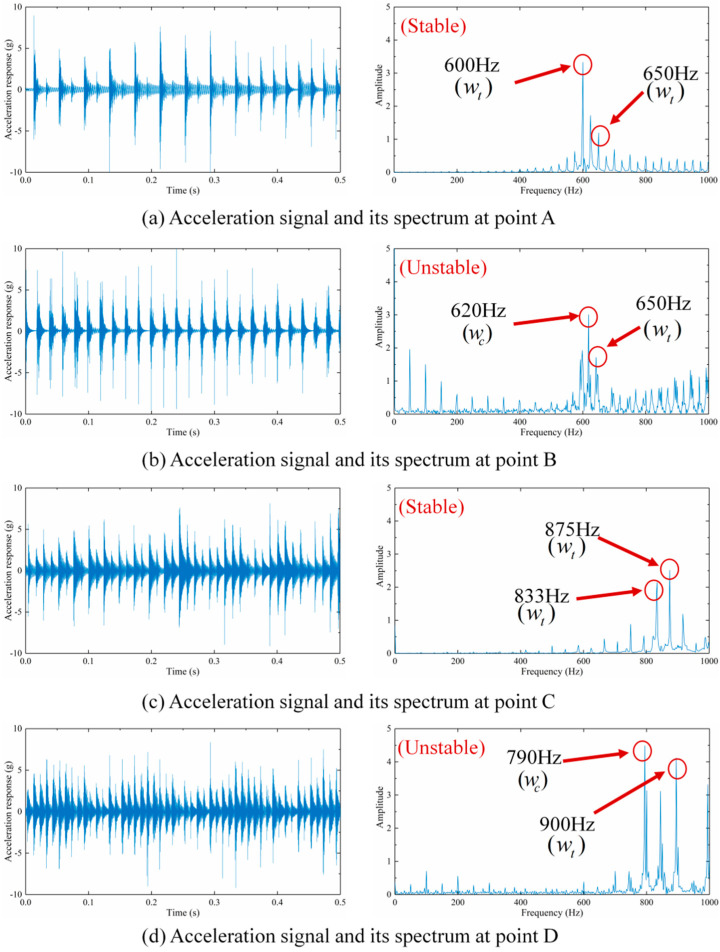
Acceleration signal and its spectrum at point A, B, C, and D in the milling of the thin-walled plate.

In this section, in order to validate the accuracy of the proposed approach for quickly and accurately predicting the stability of the milling system, the four degree of freedom in the X and Y direction for the milling cutter and workpiece in the X and Y direction for thin-walled section are considered. According to the proposed method, the stability lobe diagram with the discrete number *m* = 40 at the *a/D* = 0.1 is calculated, and the milling parameters are determined based on the stability lobe diagram as shown in Figure 8. The milling parameters in points A(*n* = 1500 rpm, *a_p_* = 0.2 mm) and C(*n* = 2500 rpm, *a_p_* = 0.4 mm) are stable parameters, while the points B(*n* = 1500 rpm, *a_p_* = 0.6 mm) and D(*n* = 3000 rpm, *a_p_* = 0.4 mm) are located in the unstable cutting region. All the dynamic responses in different points are measured and investigated, and only the dynamic response and its spectrum in points A, B, C, and D are shown in Figure 9. From Figure 9a,c, it can be seen that there is only the tool tooth passing frequency (i.e., 200 Hz, 400 Hz, 600 Hz, 650 Hz, 666 Hz, 833 Hz, 875 Hz, and 917 Hz.). From Figure 9b,d, it can be seen that the chatter frequency (i.e., 520 Hz, 580 Hz, 620 Hz, 660 Hz, 690 Hz, 790 Hz, 860 Hz, and 910 Hz.) occurs besides at the tool tooth passing frequency (i.e., 100 Hz, 200 Hz, 300 Hz, 400 Hz, 500 Hz, 600 Hz).

**Figure 10:**
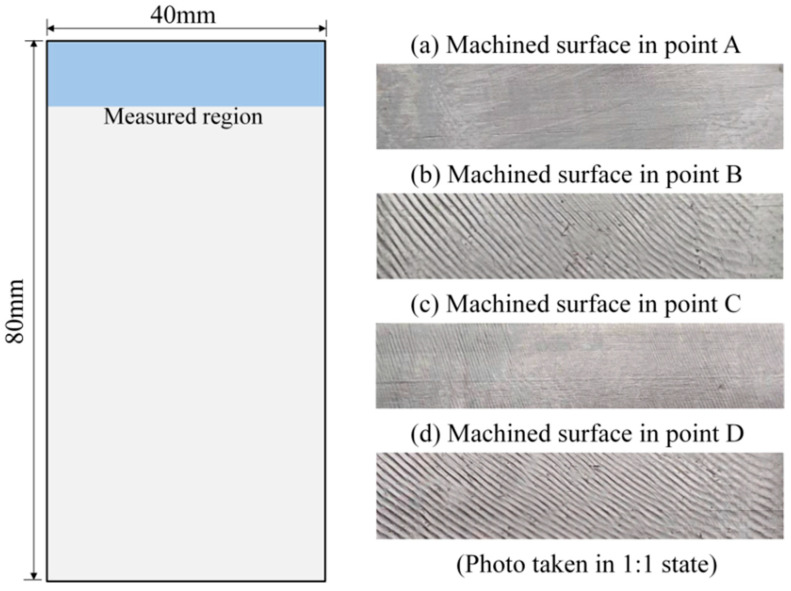
Surface morphology of the thin plate in the milling region.

In addition, in order to more clearly investigate the milling chatter, the morphologies of the machined surface at different points are shown in Figure 10. From Figure 10, we can see that the machining chatter occurs at observation points B and D, which can be observed from the rough surface quality and obvious vibration.

## 7. Conclusions

(1) A novel updated FDM is proposed to predict the milling SLD. The cubic-spline interpolation and the Newton interpolation are introduced to approximate the state item and time-delay item, respectively. A discrete map is established between the current state matrix and the previous state matrix, and the SLD is obtained based on the eigenvalue modulus judgement criterion of the transition matrix.

(2) An iterative algorithm is proposed to obviously improve computational efficiency. The calculation of the transition matrix eigenvalues in the chattering region is eliminated. The simulation results of a benchmark example with two different radial immersion ratios show that the algorithm has a faster computational efficiency than other methods, especially when the radial immersion ratio is large.

(3) The proposed method has obvious advantages in terms of computational accuracy and convergence speed than other methods. In terms of calculation accuracy, it already coincides with the reference curve when the discrete number *m* is small whether the radial immersion ratio is large or small. In addition, it has a faster convergence speed both in the stable or unstable region, and this part will be further studied in the future.

(4) A series of milling experiments under different spindle speeds are designed to verify the accuracy of the proposed method. The experimental results show that the proposed method is in good agreement with the experimental value.

## Figures and Tables

**Table 1:** The properties of the workpiece and the specifications of the cutter.

Cutter	Diameter (mm)	Number of Flutes	Helix Angle (°)	Length (mm)
	12	2	30	75
Workpiece	Density (g/cm^3^)	Possion’s Ration	Young’s Modulus (GPa)	Material
	4.6	0.34	108	Ti-6Al-4V

**Table 2:** Modal parameters of the cutter and workpiece.

System	Natural Frequency ω (Hz)	Damping Ratio ζ	Stiffness k (N∙m^−1^)
Workpiece (Mode no.1)	575	0.007	1.19 × 10^6^
Workpiece (Mode no.2)	1820	0.012	1.31 × 10^7^
Cutter in X direction	2126	0.036	1.45 × 10^8^
Cutter in Y direction	2134	0.033	1.47 × 10^8^
